# Altered m^6^A modification is involved in up‐regulated expression of *FOXO3* in luteinized granulosa cells of non‐obese polycystic ovary syndrome patients

**DOI:** 10.1111/jcmm.15807

**Published:** 2020-09-01

**Authors:** Shen Zhang, Wenli Deng, Qiongyou Liu, Peiyu Wang, Wei Yang, Wuhua Ni

**Affiliations:** ^1^ Reproductive Medicine Center The First Affiliated Hospital of Wenzhou Medical University Wenzhou China; ^2^ Department of Ophthalmology The First Affiliated Hospital of Chongqing Medical University Chongqing China; ^3^ School of Basic Medical Sciences Zunyi Medical University Zunyi China; ^4^ The State Key Laboratory of Respiratory Disease Guangdong Provincial Key Laboratory of Allergy & Clinical Immunology The Second Affiliated Hospital Guangzhou Medical University Guangzhou China; ^5^ Present address: Department of Obstetrics and Gynecology Reproductive Medicine Center The Second Affiliated Hospital Chongqing Medical University Chongqing China

**Keywords:** FOXO3, luteinized granulosa cells, mRNA decay, N^6^‐methyladenosine, polycystic ovary syndrome

## Abstract

The pathophysiology of polycystic ovary syndrome (PCOS) is characterized by granulosa cell (GC) dysfunction. m^6^A modification affects GC function in patients with premature ovarian insufficiency (POI), but the role of m^6^A modification in PCOS is unknown. The purpose of the prospective comparative study was to analyse the m^6^A profile of the luteinized GCs from normovulatory women and non‐obese PCOS patients following controlled ovarian hyperstimulation. RNA m^6^A methylation levels were measured by m^6^A quantification assay in the luteinized GCs of the controls and PCOS patients. Then, m^6^A profiles were analysed by methylated RNA immunoprecipitation sequencing (MeRIP‐seq). We reported that the m^6^A level was increased in the luteinized GCs of PCOS patients. Comparative analysis revealed differences between the m^6^A profiles from the luteinized GC of the controls and PCOS patients. We identified *FOXO3* mRNA with reduced m^6^A modification in the luteinized GCs of PCOS patients. Selectively knocking down m^6^A methyltransferases or demethylases altered expression of FOXO3 in the luteinized GCs from the controls, but did not in PCOS patients. These suggested an absence of m^6^A‐mediated transcription of *FOXO3* in the luteinized GCs of PCOS patients. Furthermore, we demonstrated that the involvement of m^6^A in the stability of the *FOXO3* mRNA that is regulated via a putative methylation site in the 3’‐UTR only in the luteinized GCs of the controls. In summary, our findings showed that altered m^6^A modification was involved in up‐regulated expression of *FOXO3* mRNA in the luteinized GCs from non‐obese PCOS patients following controlled ovarian hyperstimulation.

## 1 INTRODUCTION

Polycystic ovary syndrome (PCOS) is a common endocrine disorder and a common cause of female infertility in women of reproductive age.^1^ One of the main characteristics of the syndrome is granulosa cell (GC) dysfunction, which largely contributes to hyperandrogenism, abnormal follicle development and anti‐Mullerian hormone excess.^2^ Aberrant gene expression profile was found in GCs of PCOS patients.^3^ Multiple differential expressed genes were high related to the pathogenesis of PCOS. However, the regulation mechanisms of these genes were largely unknown.

Forkhead Box O3 (FOXO3) plays important roles in diverse cellular processes including apoptosis, metabolism, cell proliferation and cell survival.^4^ FOXO3 is regulated at several mechanistic levels, such as transcriptional activity, cellular localization, mRNA expression and protein stability. Oxidative stress induces FOXO activation and nuclear translocation by c‐Jun N‐terminal kinase (JNK) or mammalian Ste20‐like kinase 1 (MST1) activation despite phosphorylation by protein kinase B (Akt).^5^, ^6^ After energy deprivation, the increased AMP/ATP ratio leads to AMP‐activated protein kinase (AMPK) activation.^7^ AMPK activates FOXO3 activity by phosphorylation at six different residues.^8^ Insulin‐like growth factor‐1 (IGF‐I)/insulin and phosphoinositide 3‐kinase (PI3K)/Akt signalling pathway inactivate FOXOs by phosphorylation resulting in FOXOs nuclear exclusion.^9^ Zhao et al reported that elevated wnt family member 5A (WNT5a) activates PI3K/Akt signalling in GCs of PCOS patients.^10^ By contrast, Rice and colleagues showed metabolic insulin resistance in GCs of PCOS patients, suggesting that PI3K/Akt signalling is impaired.^11^ While many studies have been focused on the regulation of FOXO3 activity by post‐translational modifications, the regulation of *FOXO3* expression is largely unknown. *FOXO3* is one of the differential expressed genes which have higher expression in PCOS.^3^ Its overexpression in GCs is associated with higher apoptosis in PCOS.^12^ To date, the factors that up‐regulate and activate FOXO3 in GCs of PCOS patients are still unclear.

m^6^A is the most prevalent modification of mRNA in higher eukaryotes.^13^ The modifications are reversible and dynamically regulated by the m^6^A modulators. m^6^A modulators consist of the ‘writers’, the ‘erasers’ and the ‘readers’. Briefly, m^6^A modifications are installed by the ‘writers’ (Methyltransferase like 3 (METTL3) and Methyltransferase like 14 (METTL14)), removed by the ‘erasers’ (Fat mass and obesity‐associated protein (FTO) and alkB homolog 5(ALKBH5)), and recognized by the ‘readers’ (YTH domain‐containing proteins and Eukaryotic initiation factor 3 (eIF3)).^14^, ^15^, ^16^, ^17^, ^18^, ^19^, ^20^, ^21^, ^22^ m^6^A modification has diverse biological functions such as nuclear RNA export, RNA splicing, protein translation regulation and RNA decay. In the YTHDF2‐mediated decay pathway, mRNAs with increased m^6^A abundance in 3’‐UTR are down‐regulated due to reduced RNA stability.^19^

Several human diseases are associated with altered m^6^A modification. m^6^A levels of sperm RNA are elevated in patients with asthenozoospermina.^23^ Increased m^6^A levels in the granulosa cells were reported in patients with premature ovarian insufficiency (POI), affecting apoptosis and cell proliferation in GCs.^24^ Aberrant m^6^A modification, through the effects on RNA metabolism, plays critical roles in a variety of cancers.^25^ However, whether m^6^A modification plays a role in the pathogenesis of PCOS is unknown.

Given that m^6^A modification affects the GC function in patients with POI,^24^ we suggested alteration of the m^6^A profile in the luteinized GCs of PCOS patients, which may account for the dysregulation of certain key genes for PCOS. Here, we showed the differences of m^6^A distribution between the luteinized GCs of normovulatory women and PCOS patients following controlled ovarian hyperstimulation. We identified *FOXO3* mRNA with differential m^6^A peaks, targeted for decay by YTHDF2. Our results indicated that hypomethylated *FOXO3* mRNA caused the dysregulation of *FOXO3* in luteinized GCs from PCOS patients following controlled ovarian hyperstimulation.

## 2 SUBJECTS AND METHODS

### 2.1 Subjects

Forty‐three control patients with tubal factor infertility or male infertility and 36 PCOS patients were recruited in the Reproductive Medicine Center at the First Affiliated Hospital of Wenzhou Medical University between December 2017 and August 2019. Inclusion criteria for control patients were aged between 25 and 35 years, having a regular menstrual cycle, serum testosterone (T) level < 2 nmol/L, 18.5 < body mass index (BMI) < 27, having a normal ovarian reserve and a normal uterus. PCOS was diagnosed according to the Rotterdam revised criteria.^26^ The inclusion criteria for PCOS patients were aged between 25 and 35 years, having oligo‐ or anovulation, serum testosterone level > 2 nmol/L, 18.5 < BMI < 27, anti‐Müllerian hormone (AMH) > 7 ng/mL and having a normal uterus. The exclusion criteria were smoking, systemic diseases, endometriosis, abnormal serum level of prolactin, dysfunctional thyroid or having previous long‐term medication use.

This study was approved by the Ethics Committees of the First Affiliated Hospital of Wenzhou Medical (Approved number: YS2016‐063 and YS2019‐046). Written informed consent was obtained from all the participants.

### 2.2 Measurement of hormones

Plasma levels of AMH, luteinizing hormone (LH), follicle‐stimulating hormone (FSH), T and estradiol (E2) were collected and measured by chemiluminescence immunoassay (CLIA) between day 3 and day 5 of the menstrual cycle before the controlled ovarian stimulation. Serum fasting glucose levels and fasting insulin levels were measured by an oxidase‐peroxidase method and a CLIA method, respectively. The inter‐assay coefficients of variation were 6.1% for AMH, 7.2% for LH, 5.3% for FSH, 10.0% for T, 8.9% for E2, 4.3% for glucose and 5.4% for insulin. The intra‐assay coefficients of variation were 3.8% for AMH, 5.3% for LH, 4.6% for FSH, 8.1% for T, 6.8% for E2, 2.1% for glucose and 2.9% for insulin. Insulin in follicle fluids was detected by enzyme‐linked immunosorbent assay (ELISA, R&D Systems). HOMA‐IR was calculated by the formula (HOMA‐IR = fasting insulin (mIU/L) × fasting glucose (mmol/L)/ 22.5).^27^

### 2.3 Transvaginal ultrasonography

Ultrasound examination was performed on the 3rd‐5th day of the menstrual cycle with a 7 MHz transvaginal transducer (LOGIC 400, General Electric Medical Systems) to calculate follicle number. The basal antral follicle count (AFC) was assessed as the sum of all follicles of 2‐10 mm in diameter. The polycystic ovary was defined as 12 or more AFC in each ovary.

### 2.4 Ovarian stimulation and GCs isolation

All participants underwent controlled ovarian stimulation with gonadotrophin‐releasing hormone (GnRH) agonist long protocol. Briefly, patients received 0.1 mg/day of GnRH agonist (Decapeptyl, Ferring, Germany) from day 20 of a spontaneous menstrual cycle until the day of human chorionic gonadotrophin (hCG) injection. When pituitary down‐regulation was achieved (usually after 14 days of GnRH agonist injection), ovarian stimulation was initiated with a minimum of 150 IU/day of recombinant human FSH (rhFSH, GonalF, Merck Serono). The dosages of rhFSH were adjusted according to serum E2 levels and follicle growth of the patients. 250 µg of recombinant hCG (Ovidrel, Merck Serono) was administered, when at least three follicles were >17 mm in diameter. Transvaginal aspiration was performed 34‐36 hours later to retrieve oocytes and follicular fluids. The follicular fluids from follicles >14 mm in diameter and without obvious blood contamination were collected. The follicular fluids were centrifuged at 340 g for 8 minutes to pellet the GCs. The cell pellets were resuspended in 1× PBS solution (Gibco), overlaid on 40%/80% gradient solution (PureCeption, SAGE) and centrifuged at 320 g for 20 minutes. GCs in the interface were collected and washed with 1× PBS solution.

### 2.5 RNA m^6^A quantification

Total RNA was isolated using the Total RNA Kit II Kit (Omega Bio‐Tek). Polyadenylated RNA was purified from total RNA using the GenElute mRNA Miniprep Kit (Sigma). 500 ng total RNA or polyadenylated RNA was used to determine the RNA m^6^A methylation levels using the EpiQuik m^6^A RNA Methylation Quantification Kit (Epigentek). Briefly, a standard curve was prepared according to the manufacturer's instruction. The RNA samples were coated on the strip wells, followed by incubation with capture antibody. After washes with washing buffer, detection antibody and enhance solution were added separately. Then, the signals were developed by the detection solution. The RNA m^6^A levels were quantified by the absorbance at 450 nm, and the m^6^A contents were calculated based on the standard curve.

### 2.6 Methylated RNA immunoprecipitation sequencing (MeRIP‐seq) and MeRIP‐qPCR

MeRIP was performed using Magna MeRIP m^6^A Kit (Millipore, Billerica, MA, USA) following the manufacturer's instruction. Briefly, 50 µg of total RNA was precipitated using 2.5 volumes of 100% ethanol, one‐tenth volumes of 3 mol/L sodium acetate and 1 mg/mL glycogen. Then, the RNA was resuspended, fragmented and immunoprecipitated with anti‐m^6^A antibody or normal mouse IgG. 10% of fragmented RNA was keep as input. The immunoprecipitated RNA was eluted by competition with m^6^A sodium salt and recovered using miRNeasy Mini Kit (QIAGEN, Hilden, Germany).

The recovered RNA was treated with DNase I (0.1 U/µL, Thermo Scientific, Waltham, MA, USA) and reverse‐transcribed by random hexamer primer and RevertAid Reverse Transcriptase (Thermo Scientific). Subsequently, second strand cDNA was synthesized using DNA Polymerase I and RNase H (Thermo Scientific). Sequencing library was constructed using NEBNext Ultra Directional RNA Library Prep Kit for Illumina (NEB) and purified using AMPure XP system (Beckman Coulter). Library quality was assessed on a Bioanalyzer 2100 (Agilent). The clustering of the index‐coded samples was generated on a cBot Cluster Generation System using TruSeq PE Cluster Kit v3‐cBot‐HS (Illumina). Library preparations were sequenced on a Hiseq 4000 Platform (Illumina).

Real‐time quantitative RT‐PCR analysis (qRT‐PCR) was carried out with the samples with anti‐m^6^A antibody, the samples with normal IgG, and input samples in triplicates. Ct of the samples with anti‐m^6^A antibody were normalized to input by subtracting the Ct of input from the Ct of IP samples: ∆Ct = Ct_IP_‐(Ct_input_‐Log2[Input Dlilution Factor]). Then, the per cent of input for each IP sample was calculated: % Input = 2^−ΔCt(normalized IP)^. The primers for MeRIP‐qPCR were listed in Table S1.

### 2.7 MeRIP‐seq data analysis

Sequencing data were mapped to the reference genome (ftp://ftp.ensembl.org/pub/release‐94/fasta/homo_sapiens/dna/) using BWA mem (version 0.7.12). m^6^A peaks were identified by peak finding algorithm in MACS2 (version 2.1.0). The threshold of enrichment was set at *q* < 0.05. Differential peak analysis was based on the fold enrichment of peaks between the PCOS group and the control group. When the odds ratio > 2, a differential peak was determined. The statistics of pathway enrichment of genes with differential peaks in KEGG pathway was tested using KOBAS software.

### 2.8 Primary cell culture and RNA interference

The purified luteinized GCs were cultured in high‐glucose Dulbecco's modified Eagle's medium (DMEM, Gibco) containing 10% foetal bovine serum (FBS, Gibco) and 1% penicillin‐streptomycin (Gibco) at 37°C in a humidified incubator under 5% CO_2_. The siRNAs against YTHDF2, METTL3, METTL14, FTO and ALKBH5 were obtained from GenePharma Corporation (China). When the confluence reached 80%, the cells were dissociated and seeded in 24‐well plates (10^5^ cells/well). One day later, the cells were transfected with siRNA (GenePharma) targeting negative control, YTHDF2, METTL3, METTL14, FTO or ALKBH5 using Lipofectamine 2000 (Invitrogen) according to the manufacturer's instruction. The sequences of siRNA were listed in Table S2.

### 2.9 Real‐time quantitative RT‐PCR analysis

For the GCs collected from follicular fluids, total RNA was extracted using the Total RNA Kit II Kit (Omega Bio‐Tek). For the GCs cultured in 24‐well plates, total RNA in each well was extracted using MicroElute Total RNA Kit (Omega Bio‐Tek). The total RNA was reverse‐transcribed by the PrimeScript RT reagent Kit with gDNA Eraser (Takara) according to the manufacturer's instruction. Real‐time quantitative RT‐PCR analysis (qRT‐PCR) was performed on a Corbett Rotor Gene 6000 Real‐time Cycler using TB Green Premix Ex Taq II (Takara). Each sample was analysed in triplicates to obtain the threshold cycle number. *GAPDH* mRNA was used as an internal control for normalization. Relative expression levels were quantified using comparative 2^‐∆∆Ct^ method and expressed as fold changes relative to the controls. The sequences of the primers were listed in Table S1.

### 2.10 Western blot analysis

The total protein was extracted from the cultured GCs using RIPA buffer (Beyotime) containing 1mM PMSF (Beyotime). The concentration of the protein was measured using BCA protein assay kit (Solarbio). The cell extracts were separated on a 12% SDS‐PAGE Gel (ExpressPlus PAGE Gel, GenScript) and transferred to methanol‐activated polyvinylidenefluoride membranes. The membranes were blocked with 5% non‐fat milk powder for 1 hour and incubated with the primary antibody for 2 hour at room temperature. The mouse anti‐FOXO3 (Proteintech, 66428‐1‐Ig) or mouse anti‐Beta‐actin (Proteintech, 60008‐1‐Ig) antibody was used as primary antibody. Then, the membranes were incubated with HRP‐conjugated affinipure goat anti‐mouse IgG (H + L) (Biosharp, BL1001A) for 1 hour at room temperature. The signals were developed with SuperSignal West Pico PLUS Chemiluminescent Substrate (Thermo Scientific).

### 2.11 Plasmid construction and dual‐luciferase reporter assay

The proximal 3’‐UTR of *FOXO3* was amplified and inserted into 3’‐downstream of the *Renilla* luciferase gene of the psiCHECK^TM^‐2 Vector between XhoI and NotI restriction sites. The motif of m^6^A binding was mutated by fusion PCR with a pair of complementary primers containing the A‐to‐T mutation. The primary culture human GCs were transfected with wild‐type m^6^A motif reporter plasmid or mutant m^6^A motif reporter plasmid using Lipofectamine 2000. The *Firefly* and *Renilla* luciferase activities were measured using the Dual‐Luciferase Reporter Assay System (Promega) 48 hours later. The *Renilla* luciferase activities were normalized to *Firefly* luciferase activities.

### 2.12 Statistical analysis

Data are expressed as the means ± SD. The normality and homogeneity of variance of the data were assessed. Differences between groups were determined by one‐way analysis of variance (ANOVA) followed by the Tukey multiple comparison test using SPSS 16.0 software package. Significance was set at *P* < 0.05.

## 3 RESULTS

### 3.1 m^6^A levels in the luteinized GCs of the controls and PCOS patients

**Figure 1:**
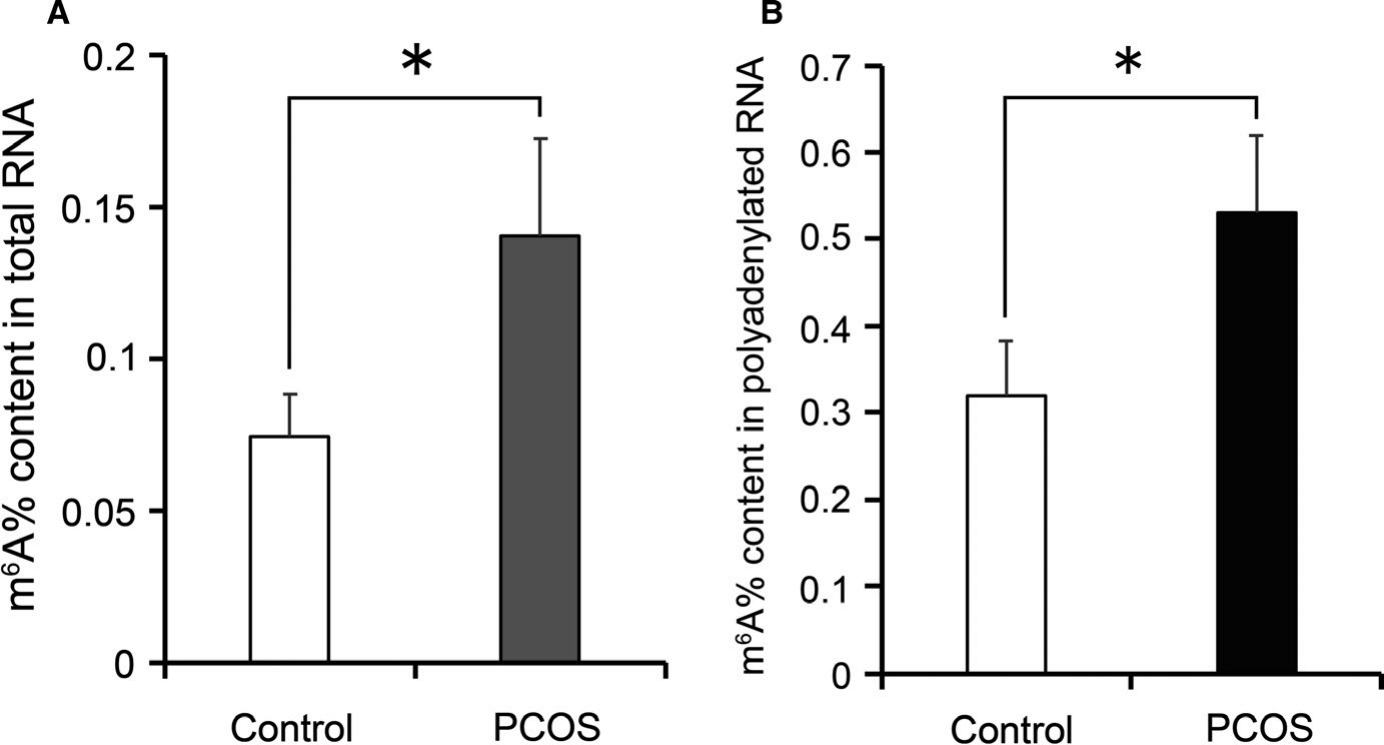
Quantification analysis of m^6^A modification in the luteinized GCs of the controls and PCOS patients. The m^6^A content in total RNA (A) and mRNA (B) of GCs were detected by colorimetric assay. Bars represent means ± SD, n = 5. *P < 0.05 vs the control

To investigate the role of m^6^A modification in the luteinized GCs of PCOS patients, we recruited normovulatory controls and PCOS patients in our reproductive centre (Table S3). The insulin‐glucose parameters indicated that the PCOS patients had insulin resistance in the present study. We examined m^6^A levels in the total RNA of the luteinized GCs collected following ovarian hyperstimulation by RNA m^6^A quantification. m^6^A levels were twofold higher in the luteinized GCs of PCOS patients compared with the controls (Figure 1A). We further isolated polyadenylated RNA from the total RNA and examined the m^6^A levels. Consistently, the m^6^A levels of polyadenylated RNA were increased in the luteinized GCs of PCOS patients (Figure 1B).

### 3.2 Differential m^6^A modification in the luteinized GCs of the controls and PCOS patients

**Figure 2:**
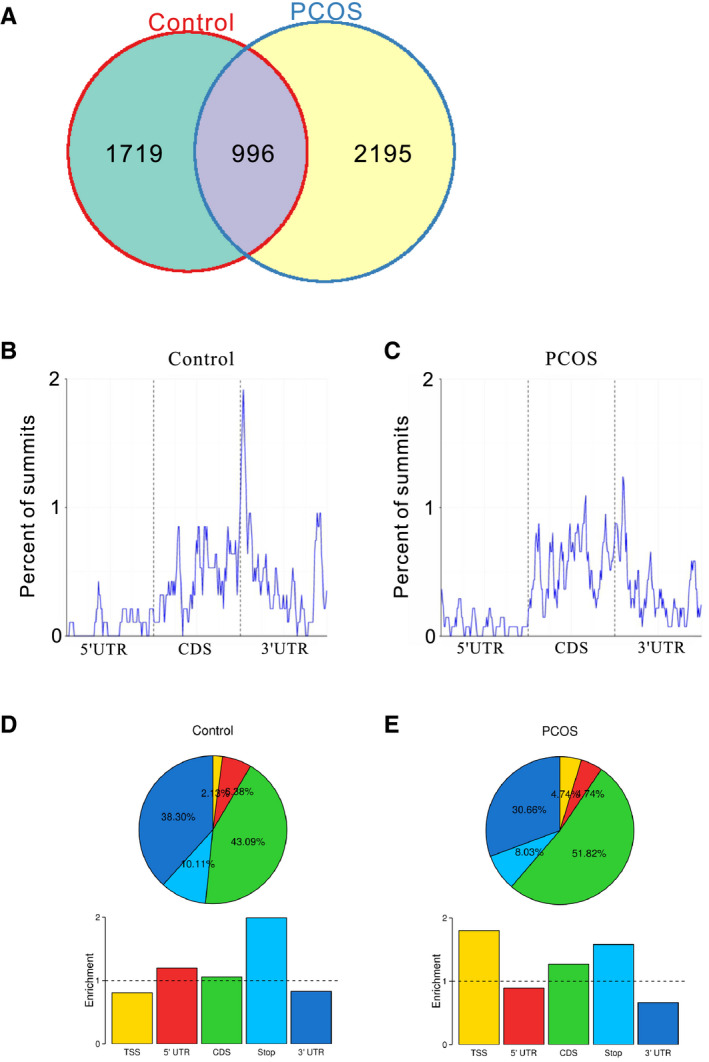
Distribution of m^6^A peaks in the luteinized GCs of the controls and PCOS patients. A, Comparison of the m^6^A peaks between the controls and PCOS patients. (B and C) Metagene profiles of enrichment of m^6^A peaks across mRNA transcriptome of the controls (B) and PCOS patients (C). (D and E) Distribution and enrichment of the m^6^A peaks within different gene regions in the GCs of the controls (D) and PCOS patients (E). TSS, transcription start sites; 5’‐UTR, 5’‐untranslated region; CDS, coding sequence; Stop, translation termination sites; 3’‐UTR, 3’‐untranslated region

We conducted MeRIP‐seq to analyse the transcriptome‐wide distribution of m^6^A modification in GCs. 2764 and 3405 m^6^A peaks were identified in the controls and PCOS patients, respectively (Table S4). We selected 8 transcripts with m^6^A peaks to verify the sequencing data by MeRIP‐qPCR. The qRT‐PCR results were in agreement with the sequencing data (Figure S1). We analysed the differential peaks based on the fold enrichment of peaks of the two groups. 1719 and 2195 peaks were distinct in the controls and PCOS patients, respectively, while 996 peaks were overlapped in both groups (Figure 2A and Table S5).

Similar to the other studies, m^6^A peaks were strongly enriched around the stop codon in the controls (Figure 2B,D).^28^, ^29^, ^30^ However, m^6^A peaks in PCOS patients showed a less prominent enrichment around the stop codon and increased locations to the CDS (coding sequence) and the TSS (transcription start sites) regions (Figure 2C,E).

### 3.3 m^6^A modification targets the *FOXO3* transcript in the luteinized GCs of the controls but not PCOS patients

**Figure 3:**
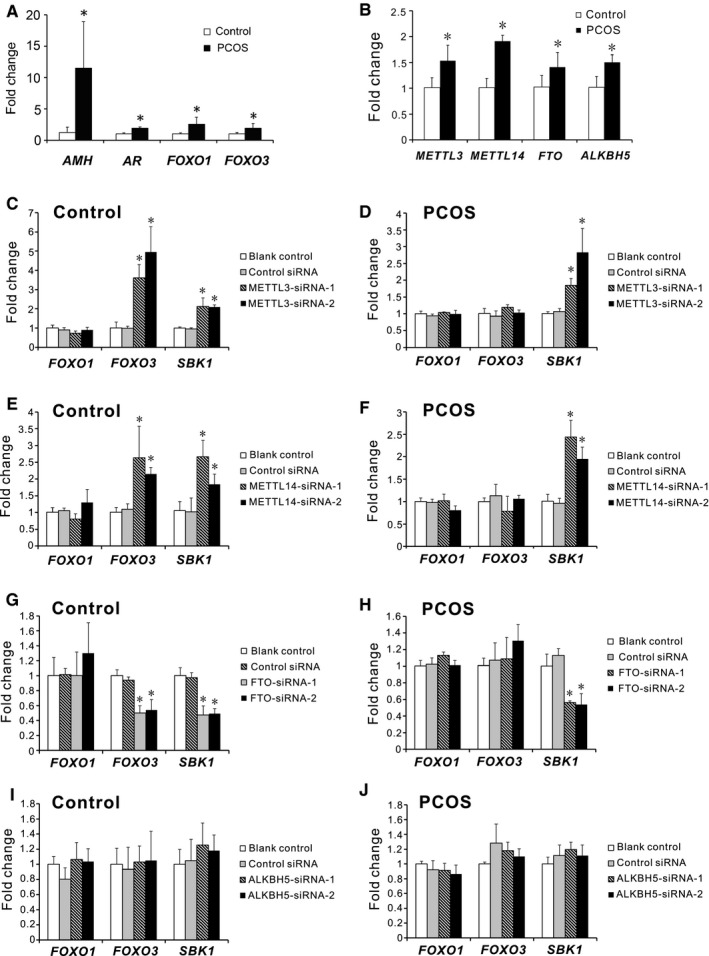
Involvement of m^6^A modification in regulation of *FOXO3* mRNA in the luteinized GCs of the controls but not PCOS patients. A, mRNA levels of PCOS‐associated genes (*AMH*, *AR*, *FOXO1* and *FOXO3*) in the luteinized GCs from the controls and PCOS patients. B, Expression of the methyltransferases (*METTL3* and *METTL14*) and demethylases (*FTO* and *ALKBH5*) in the luteinized GCs of controls and PCOS patients. C‐J, The effects of selectively knockdown of the methyltransferases or demethylases on the expression of *FOXO1*, *FOXO3* and *SBK1* in the luteinized GCs of the controls and PCOS patients. GCs of the controls and PCOS patients were collected, cultured and subjected to METTL3‐siRNA (C,D), METTL14‐siRNA (E,F), FTO‐siRNA (G,H) or ALKBH5‐siRNA (I,J), respectively. qRT‐PCR analysis was performed to examine the mRNA levels of *FOXO1*, *FOXO3* and *SBK1*. Data were expressed as fold changes relative to the blank controls. Bars represent means ± SD, n = 5. *P < 0.05 vs the blank controls

We suggested that m^6^A modification plays a role in dysregulation of PCOS‐associated genes. We examined the expression of *AMH*, *AR*, *FOXO1* and *FOXO3* in our study. Consistent with the previous studies,^2^, ^3^, ^31^ the expression of *AMH*, *AR*, *FOXO1* and *FOXO3* were up‐regulated in the luteinized GCs of PCOS patients compared with the controls (Figure 3A). To screen potential candidates, we compared the expression of the m^6^A methyltransferases and demethylases in the luteinized GCs of the controls and PCOS patients. The expression of *METTL3*, *METTL14*, *FTO* and *ALKBH5* were all elevated in PCOS patients compared with the controls (Figure 3B). We selectively knocked down the expression of *METTL3*, *METTL14*, *FTO* or *ALKBH5* in the luteinized GCs of the controls and PCOS patients, respectively. The knockdown efficiency was confirmed by qRT‐PCR analysis (Figure S2). Due to differential m^6^A peaks identified in *FOXO1* and *FOXO3* transcripts, we examined the expression of *FOXO1* and *FOXO3* in these cells. In the controls, depletion of METTL3 or METTL14 increased the expression of *FOXO3*, and silencing of FTO suppressed the expression of *FOXO3*, suggesting m^6^A modification regulated *FOXO3* mRNA degradation (Figure 3C,E,G). However, depletion of the m^6^A methyltransferases or demethylases did not affect the expression of *FOXO3* in PCOS patients (Figure 3D,F,H), which indicated that effects of m^6^A modification on *FOXO3* mRNA were cell‐specific. Our results also showed that knockdown of methyltransferases or demethylases did not affect the expression of *FOXO1* in GCs (Figure 3C‐J). The common target gene *SBK1* was used as a positive control to confirm the results. Collectively, these results demonstrated that selectively knocking down m^6^A methyltransferases or demethylases did not alter the expression of *FOXO3* in the luteinized GCs of PCOS patients, but did so in the controls, suggesting an absence of m^6^A‐regulated transcription of *FOXO3* in the luteinized GCs of PCOS patients.

### 3.4 m^6^A modification regulates the stability of the *FOXO3* transcript via the YTHDF2‐mediated decay pathway in the luteinized GCs of the controls

**Figure 4:**
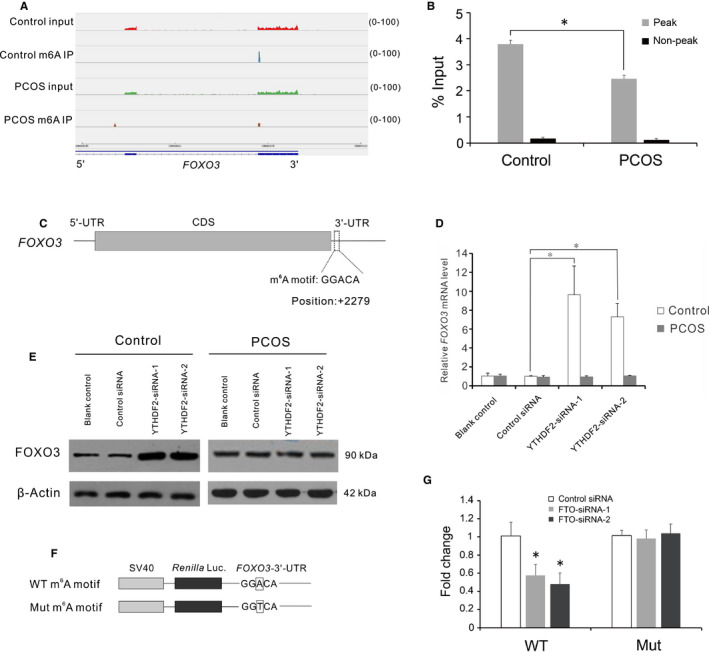
Differentially methylated *FOXO3* transcript was regulated via the YTHDF2‐mediated decay pathway in the luteinized GCs of the controls. A, m^6^A peaks distribution across 3’‐UTR of the *FOXO3* transcript. The Y‐axis shows read number. Blue boxes indicate exons, and blue line indicates introns. B, qRT‐PCR analysis of m^6^A peak region and non‐peak region in the 3’‐UTR of the *FOXO3* transcript. Bars represent means ± SD, n = 5. *P < 0.05 for the differences between the indicated groups. C, Schematic representation of the position of m^6^A motif in the 3’‐UTR of the *FOXO3* transcript. D, The mRNA level of *FOXO3* in the YTHDF2‐knockdown cells. Bars represent means ± SD, n = 5. *P < 0.05 for the differences between the indicated groups. E, Western blot analysis of FOXO3 total protein level in the YTHDF2‐knockdown cells. F, Wild‐type or m^6^A motif mutant (A‐to‐T mutation) FOXO3‐3’‐UTR fused with *Renilla* luciferase reporter. G, Relative luciferase activity of the FOXO3‐3’‐UTR with wild‐type or mutant m^6^A motif after cotransfection with negative control siRNA or FTO‐siRNA *Renilla* luciferase activity was normalized to firefly luciferase activity. Bars represent means ± SD, n = 3. *P < 0.05 vs the control siRNA group

Our MeRIP‐seq showed a differential m^6^A peak in 3’‐UTR and near the stop codon of the *FOXO3* transcript (Figure 4A). We performed MeRIP‐qPCR to confirm the differential m^6^A levels on the m^6^A target site (Figure 4B). To determine how m^6^A modification regulates the *FOXO3* transcript, we analysed the sequence of the m^6^A peak. A putative m^6^A site was identified within the m^6^A peak of 3’‐UTR at position + 2279 (+1 relative to the translation start site) (Figure 4C). As YTHDF2 is the main binding protein that account for the decay of m^6^A‐modified mRNAs, we next examined the effects of YTHDF2‐knockdown on *FOXO3* expression in human GCs. Depletion of YTHDF2 increased the amount of *FOXO3* transcript, and total protein levels of FOXO3 (Figure 4D,E). The knockdown efficiency was checked by qRT‐PCR (Figure S2). To assess the functionalities of the m^6^A site in the 3’‐UTR of *FOXO3* transcript, we constructed a reporter plasmid bearing FOXO3‐3’‐UTR with the putative m^6^A site mutated (Figure 4F). FTO‐siRNA suppress the luciferase activities of the reporter plasmid with the wild‐type m^6^A site (Figure 4G). However, mutation of the m^6^A site reversed the suppressive effects of FTO‐siRNA on the luciferase activities (Figure 4G). The presence of the m^6^A readers in human GCs was showed by a previous study.^32^ To exclude the potential of lacking the m^6^A readers in GCs of PCOS patients, we examined the expression of the m^6^A readers in PCOS. The expression of *YTHDF2* was significantly increased in the luteinized GCs of PCOS patients (Figure S3). These results indicated that m^6^A modification regulated *FOXO3* mRNA decay through the m^6^A site in 3’‐UTR in the luteinized GCs of the controls.

## 4 DISCUSSION

Increasing evidence showed that m^6^A modification play important roles in various physiological processes.^17^, ^33^, ^34^, ^35^, ^36^ Elevated or decreased m^6^A level was associated with various human diseases.^23^, ^24^, ^25^ We showed that the level of m^6^A modification was higher in the luteinized GCs of PCOS patients than that in the controls. Aberrant m^6^A levels affect gene expression and biological functions. However, whether human diseases alter m^6^A profiles is largely unknown. Here, we showed that not only elevated m^6^A level but also altered m^6^A profile are associated with PCOS in the luteinized GCs.

The site selection of m^6^A modification is largely unknown. miRNAs regulate m^6^A formation at corresponding target sites.^37^ Altered miRNAs and other associated factors partially explain the differences of m^6^A profiles among cell types.^37^ Furthermore, m^6^A profile was altered when cells exposed to heat shock, ultraviolet radiation or signalling molecules.^38^ Acute stress regulates a fraction of genes with m^6^A modification in the cortex.^33^ The GCs of PCOS patients present different cell characteristics. Cell survival, proliferation rate and responsiveness to FSH were altered in the GCs of PCOS patients.^39^, ^40^ Differentially expressed miRNAs were also identified in the GCs of PCOS patients.^41^, ^42^ Moreover, abnormal serum AMH, androgen, activin A and follistatin were observed in PCOS patients.^2^, ^43^, ^44^ All these alterations may account for the altered m^6^A profile in PCOS. We identified 2195 differential m^6^A peaks in the luteinized GCs of PCOS patients compared to those of the controls. KEGG analysis showed that differential peaks were enriched in metabolic pathway (Figure S4). Whether m^6^A modification is involved in the regulation of these transcripts with the differential m^6^A modification needs further investigation.

Insulin resistance is prevalent in PCOS patients and plays a crucial role in metabolic abnormalities. Although obesity increases risks for insulin resistance, several studies have demonstrated that both obese and non‐obese PCOS patients had impaired insulin signalling.^45^, ^46^ In the present study, the non‐obese PCOS patients had significantly higher insulin 0, insulin 120 and HOMA‐IR compared with the controls. Moreover, the insulin levels in follicular fluid were notably higher in the non‐obese PCOS patients. These indicated that the PCOS patients in our study had insulin resistance. As FOXO proteins mediate the effects of insulin signalling on metabolism,^47^ it is likely that altered expression of *FOXO3* is involved in the defects of insulin signalling in the luteinized GCs of PCOS patients. Here, we demonstrated an absence of m^6^A‐mediated *FOXO3* mRNA destabilization which altered expression of *FOXO3* in the luteinized GCs of PCOS patients. Our findings have offered a potential mechanism for insulin resistance.

It is well known that insulin activates PI3K/Akt signalling to induce the nuclear exclusion of FOXO3.^9^ However, the effects of PI3K/Akt signalling on GCs of PCOS patients are controversial. Zhao et al showed that WNT5a expression was elevated in GCs of PCOS patients.^10^ Although they have demonstrated that WNT5a increased inflammation and oxidative stress via PI3K/Akt/NF‐κB signalling in human GCs, they did not confirm that WNT5a activated PI3K/Akt/NF‐κB signalling in GCs of PCOS patients. As insulin resistance is attributed to defects in PI3K/Akt signalling, metabolic insulin resistance in GCs of PCOS patients suggests impaired PI3K/Akt signalling.^9^, ^11^ Thus, it is possible that abnormalities in other signalling cascade rather than PI3K/Akt signalling contributed to chronic inflammation in GCs of PCOS patients. To date, the factors that up‐regulate and activate FOXO3 remain unclear in GCs of PCOS patients. Here, we demonstrated that YTHDF2 mediated *FOXO3* mRNA decay via a putative m^6^A site in the 3’‐UTR in the luteinized GCs of the controls. In contrast to the findings in the controls, although the expression of YTHDF2 was elevated in the luteinized GCs of PCOS patients, *FOXO3* mRNA was hypomethylated and irresponsive to m^6^A modification. Our results showed an absence of m^6^A‐based regulation of FOXO3 expression in the luteinized GCs of PCOS patients. Taken together, we inferred that altered m^6^A modification caused upregulation of FOXO3 in the luteinized GCs of PCOS patients following controlled ovarian hyperstimulation.

In summary, the present study provided m^6^A profiles of normovulatory women and PCOS patients in luteinized GCs following controlled ovarian hyperstimulation. We demonstrated that altered m^6^A modification disturbed the regulation of FOXO3 expression in the luteinized GCs of PCOS patients. However, the site selection mechanism of m^6^A in PCOS needs to be explored in future studies.

## CONFLICT OF INTEREST

The authors confirm that there are no conflicts of interest.

## AUTHOR CONTRIBUTION

**Shen Zhang:** Conceptualization (lead); Data curation (lead); Funding acquisition (equal); Investigation (lead); Methodology (lead); Writing‐original draft (lead); Writing‐review & editing (lead). **Wenli Deng:** Conceptualization (supporting); Data curation (supporting); Investigation (supporting); Methodology (supporting); Writing‐original draft (supporting); Writing‐review & editing (supporting). **Qiongyou Liu:** Investigation (supporting); Methodology (supporting); Writing‐review & editing (supporting). **Peiyu Wang:** Data curation (supporting); Investigation (supporting); Methodology (supporting). **Wei Yang:** Investigation (supporting); Methodology (supporting); Writing‐review & editing (supporting). **Wuhua Ni:** Funding acquisition (equal); Project administration (lead).

## Supporting information

Fig S1

Fig S2

Fig S3

Fig S4

Table S1

Table S2

Table S3

Table S4

Table S5

## References

[1] Norman RJ , Dewailly D , Legro RS , et al. Polycystic ovary syndrome. Lancet. 2007;370:685‐697. 17720020 10.1016/S0140-6736(07)61345-2

[2] Catteau‐Jonard S , Jamin SP , Leclerc A , et al. Anti‐Mullerian hormone, its receptor, FSH receptor, and androgen receptor genes are overexpressed by granulosa cells from stimulated follicles in women with polycystic ovary syndrome. J Clin Endocrinol Metab. 2008;93:4456‐4461. 18697861 10.1210/jc.2008-1231

[3] Haouzi D , Assou S , Monzo C , et al. Altered gene expression profile in cumulus cells of mature MII oocytes from patients with polycystic ovary syndrome. Hum Reprod. 2012;27:3523‐3530. 22951915 10.1093/humrep/des325

[4] Accili D , Arden KC . FoxOs at the crossroads of cellular metabolism, differentiation, and transformation. Cell. 2004;117:421‐426. 15137936 10.1016/s0092-8674(04)00452-0

[5] Sunayama J , Tsuruta F , Masuyama N , et al. JNK antagonizes Akt‐mediated survival signals by phosphorylating 14‐3‐3. J Cell Biol. 2005;170:295‐304. 16009721 10.1083/jcb.200409117 PMC2171419

[6] Lehtinen MK , Yuan Z , Boag PR , et al. A conserved MST‐FOXO signaling pathway mediates oxidative‐stress responses and extends life span. Cell. 2006;125:987‐1001. 16751106 10.1016/j.cell.2006.03.046

[7] Hardie DG , Ross FA , Hawley SA . AMPK: a nutrient and energy sensor that maintains energy homeostasis. Nat Rev Mol Cell Biol. 2012;13:251‐262. 22436748 10.1038/nrm3311 PMC5726489

[8] Greer EL , Oskoui PR , Banko MR , et al. The energy sensor AMP‐activated protein kinase directly regulates the mammalian FOXO3 transcription factor. J Biol Chem. 2007;282:30107‐30119. 17711846 10.1074/jbc.M705325200

[9] Hopkins BD , Goncalves MD , Cantley LC . Insulin‐PI3K signalling: an evolutionarily insulated metabolic driver of cancer. Nat Rev Endocrinol. 2020;16:276‐283. 32127696 10.1038/s41574-020-0329-9 PMC7286536

[10] Zhao Y , Zhang C , Huang Y , et al. Up‐regulated expression of WNT5a increases inflammation and oxidative stress via PI3K/AKT/NF‐kappaB signaling in the granulosa cells of PCOS patients. J Clin Endocrinol Metab. 2015;100:201‐211. 25303486 10.1210/jc.2014-2419

[11] Rice S , Christoforidis N , Gadd C , et al. Impaired insulin‐dependent glucose metabolism in granulosa‐lutein cells from anovulatory women with polycystic ovaries. Hum Reprod. 2005;20:373‐381. 15539436 10.1093/humrep/deh609

[12] Mikaeili S , Rashidi BH , Safa M , et al. Altered FoxO3 expression and apoptosis in granulosa cells of women with polycystic ovary syndrome. Arch Gynecol Obstet. 2016;294:185‐192. 26993517 10.1007/s00404-016-4068-z

[13] Desrosiers R , Friderici K , Rottman F . Identification of methylated nucleosides in messenger RNA from Novikoff hepatoma cells. Proc Natl Acad Sci U S A. 1974;71:3971‐3975. 4372599 10.1073/pnas.71.10.3971 PMC434308

[14] Jia G , Fu YE , Zhao XU , et al. N6‐methyladenosine in nuclear RNA is a major substrate of the obesity‐associated FTO. Nat Chem Biol. 2011;7:885‐887. 22002720 10.1038/nchembio.687 PMC3218240

[15] Zheng G , Dahl J , Niu Y , et al. ALKBH5 is a mammalian RNA demethylase that impacts RNA metabolism and mouse fertility. Mol Cell. 2013;49:18‐29. 23177736 10.1016/j.molcel.2012.10.015 PMC3646334

[16] Xiao W , Adhikari S , Dahal U , et al. Nuclear m(6)A reader YTHDC1 regulates mRNA splicing. Mol Cell. 2016;61:507‐519. 26876937 10.1016/j.molcel.2016.01.012

[17] Hsu PJ , Zhu Y , Ma H , et al. Ythdc2 is an N(6)‐methyladenosine binding protein that regulates mammalian spermatogenesis. Cell Res. 2017;27:1115‐1127. 28809393 10.1038/cr.2017.99 PMC5587856

[18] Li A , Chen Y‐S , Ping X‐L , et al. Cytoplasmic m(6)A reader YTHDF3 promotes mRNA translation. Cell Res. 2017;27:444‐447. 28106076 10.1038/cr.2017.10 PMC5339832

[19] Wang X , Lu Z , Gomez A , et al. N6‐methyladenosine‐dependent regulation of messenger RNA stability. Nature. 2014;505:117‐120. 24284625 10.1038/nature12730 PMC3877715

[20] Wang X , Zhao B , Roundtree I , et al. N(6)‐methyladenosine modulates messenger RNA translation efficiency. Cell. 2015;161:1388‐1399. 26046440 10.1016/j.cell.2015.05.014 PMC4825696

[21] Meyer K , Patil D , Zhou J , et al. 5' UTR m(6)A promotes cap‐independent translation. Cell. 2015;163:999‐1010. 26593424 10.1016/j.cell.2015.10.012 PMC4695625

[22] Roundtree IA , Evans ME , Pan T , et al. Dynamic RNA modifications in gene expression regulation. Cell. 2017;169:1187‐1200. 28622506 10.1016/j.cell.2017.05.045 PMC5657247

[23] Yang Y , Huang W , Huang J‐T , et al. Increased N6‐methyladenosine in human sperm RNA as a risk factor for asthenozoospermia. Sci Rep. 2016;6:24345. 27072590 10.1038/srep24345 PMC4829835

[24] Ding C , Zou Q , Ding J , et al. Increased N6‐methyladenosine causes infertility is associated with FTO expression. J Cell Physiol. 2018;233:7055‐7066. 29384212 10.1002/jcp.26507 PMC13482491

[25] Liu J , Harada BT , He C . Regulation of gene expression by N(6)‐methyladenosine in cancer. Trends Cell Biol. 2019;29:487‐499. 30940398 10.1016/j.tcb.2019.02.008 PMC6527461

[26] Rotterdam EA‐SPcwg . Revised consensus on diagnostic criteria and long‐term health risks related to polycystic ovary syndrome (PCOS). Hum Reprod. 2003;2004(19):41‐47. 10.1093/humrep/deh098 14688154

[27] Matthews DR , Hosker JP , Rudenski AS , et al. Homeostasis model assessment: insulin resistance and beta‐cell function from fasting plasma glucose and insulin concentrations in man. Diabetologia. 1985;28:412‐419. 3899825 10.1007/BF00280883

[28] Zhou J , Wan JI , Gao X , et al. Dynamic m(6)A mRNA methylation directs translational control of heat shock response. Nature. 2015;526:591‐594. 26458103 10.1038/nature15377 PMC4851248

[29] Lin S , Choe J , Du P , et al. The m(6)A methyltransferase METTL3 promotes translation in human cancer cells. Mol Cell. 2016;62:335‐345. 27117702 10.1016/j.molcel.2016.03.021 PMC4860043

[30] Meyer K , Saletore Y , Zumbo P , et al. Comprehensive analysis of mRNA methylation reveals enrichment in 3' UTRs and near stop codons. Cell. 2012;149:1635‐1646. 22608085 10.1016/j.cell.2012.05.003 PMC3383396

[31] Li DA , You Y , Bi F‐F , et al. Autophagy is activated in the ovarian tissue of polycystic ovary syndrome. Reproduction. 2018;155:85‐92. 29030491 10.1530/REP-17-0499

[32] Huang B , Ding C , Zou Q , et al. Cyclophosphamide regulates N6‐methyladenosine and m6A RNA enzyme levels in human granulosa cells and in ovaries of a premature ovarian aging mouse model. Front Endocrinol (Lausanne). 2019;10:415. 31316467 10.3389/fendo.2019.00415 PMC6610338

[33] Engel M , Eggert C , Kaplick PM , et al. The role of m(6)A/m‐RNA methylation in stress response regulation. Neuron. 2018;99:389‐403. 30048615 10.1016/j.neuron.2018.07.009 PMC6069762

[34] Li H‐B , Tong J , Zhu S , et al. m(6)A mRNA methylation controls T cell homeostasis by targeting the IL‐7/STAT5/SOCS pathways. Nature. 2017;548:338‐342. 28792938 10.1038/nature23450 PMC5729908

[35] Mendel M , Chen K‐M , Homolka D , et al. Methylation of structured RNA by the m(6)A writer METTL16 is essential for mouse embryonic development. Mol Cell. 2018;71(6):986‐1000.e11. 30197299 10.1016/j.molcel.2018.08.004 PMC6162343

[36] Tong J , Cao G , Zhang T , et al. m(6)A mRNA methylation sustains Treg suppressive functions. Cell Res. 2018;28:253‐256. 29303144 10.1038/cr.2018.7 PMC5799823

[37] Chen T , Hao Y‐J , Zhang Y , et al. m(6)A RNA methylation is regulated by microRNAs and promotes reprogramming to pluripotency. Cell Stem Cell. 2015;16:289‐301. 25683224 10.1016/j.stem.2015.01.016

[38] Dominissini D , Moshitch‐Moshkovitz S , Schwartz S , et al. Topology of the human and mouse m6A RNA methylomes revealed by m6A‐seq. Nature. 2012;485:201‐206. 22575960 10.1038/nature11112

[39] Guedikian AA , Lee AY , Grogan TR , et al. Reproductive and metabolic determinants of granulosa cell dysfunction in normal‐weight women with polycystic ovary syndrome. Fertil Steril. 2018;109:508‐515. 29428312 10.1016/j.fertnstert.2017.11.017 PMC5812340

[40] Das M , Djahanbakhch O , Hacihanefioglu B , et al. Granulosa cell survival and proliferation are altered in polycystic ovary syndrome. J Clin Endocrinol Metab. 2008;93:881‐887. 18073308 10.1210/jc.2007-1650 PMC2679149

[41] Xu BO , Zhang Y‐W , Tong X‐H , et al. Characterization of microRNA profile in human cumulus granulosa cells: Identification of microRNAs that regulate Notch signaling and are associated with PCOS. Mol Cell Endocrinol. 2015;404:26‐36. 25622783 10.1016/j.mce.2015.01.030

[42] Sørensen AE , Wissing ML , Englund ALM , et al. MicroRNA species in follicular fluid associating with polycystic ovary syndrome and related intermediary phenotypes. J Clin Endocrinol Metab. 2016;101:1579‐1589. 26771704 10.1210/jc.2015-3588 PMC4880172

[43] Norman RJ , Milner CR , Groome NP , et al. Circulating follistatin concentrations are higher and activin concentrations are lower in polycystic ovarian syndrome. Hum Reprod. 2001;16:668‐672. 11278215 10.1093/humrep/16.4.668

[44] Eldar‐Geva T , Spitz IM , Groome NP , et al. Follistatin and activin A serum concentrations in obese and non‐obese patients with polycystic ovary syndrome. Hum Reprod. 2001;16:2552‐2556. 11726573 10.1093/humrep/16.12.2552

[45] Dunaif A , Segal KR , Futterweit W , et al. Profound peripheral insulin resistance, independent of obesity, in polycystic ovary syndrome. Diabetes. 1989;38:1165‐1174. 2670645 10.2337/diab.38.9.1165

[46] Dunaif A , Finegood DT . Beta‐cell dysfunction independent of obesity and glucose intolerance in the polycystic ovary syndrome. J Clin Endocrinol Metab. 1996;81:942‐947. 8772555 10.1210/jcem.81.3.8772555

[47] Barthel A , Schmoll D , Unterman TG . FoxO proteins in insulin action and metabolism. Trends Endocrinol Metab. 2005;16:183‐189. 15860415 10.1016/j.tem.2005.03.010

