# Supplementary material for: Altered m6A modification is involved in up‐regulated expression of FOXO3 in luteinized granulosa cells of non‐obese polycystic ovary syndrome patients
Source: J Cell Mol Med. 2020 Sep 1;24(20):11874–82. doi: 10.1111/jcmm.15807 (PMC7578862; doi:10.1111/jcmm.15807)
Supplement: Supplementary file 5 — Table S1 [file JCMM-24-11874-s005.docx]

**Supplemental Table S1. Sequences of the primers used for qRT-PCR and cloning.**

| **Primer Name** | **Sequence (5’→3’)** |
| --- | --- |
| **Primers for qRT-PCR** |  |
| hAMH-F | CGCCTGGTGGTCCTACAC |
| hAMH-R | GAACCTCAGCGAGGGTGTT |
| hAR-F | CCTGGCTTCCGCAACTTACAC |
| hAR-R | GGACTTGTGCATGCGGTACTCA |
| hFOXO1-F | AAGAGCGTGCCCTACTTCAA |
| hFOXO1-R | CTCCCTCTGGATTGAGCATC |
| hFOXO3-F | TGTTGGTTTGAACGTGGGGA |
| hFOXO3-R | GTTTGAGGGTCTGCTTTGCC |
| hSBK1-F | CTACGAACTAGTCCGGGAGC |
| hSBK1-R | GAGGAGAGGCTGTTGGTGAT |
| hMETTL3-F | CAAGCTGCACTTCAGACGAA |
| hMETTL3-R | GCTTGGCGTGTGGTCTTT |
| hMETTL14-F | CCCATGTACTTACAAGCC |
| hMETTL14-R | TTAGCAGTGATGCCAGTT |
| hFTO-F | GGGTTCATCCTACAACGG |
| hFTO-R | ACCTGTCCACCAGATTTT |
| hALKBH5-F | GCCGTCATCAACGACTACCA |
| hALKBH5-R | CCGAATAGGCTTGAACTGGAA |
| hYTHDC1-F | TCCACGATACCAGGAAGT |
| hYTHDC1-R | ATGGTGCTGATAGTAAGGAT |
| hYTHDC2-F | TGTAGGAGAAACTGGGTC |
| hYTHDC2-R | TGTCTTTGGAGAAACCCT |
| hYTHDF1-F | GGGGAACAACATCTATCAGC |
| hYTHDF1-R | CCAATCTTCAGGCCAACC |
| hYTHDF2-F | CGTGGCTTCCAATAGTTT |
| hYTHDF2-R | CCTTGTTATCCCAAGTTCC |
| hYTHDF3-F | CAAGGCATGACTGGACTG |
| hYTHDF3-R | GGGTTTAAGTTTCGGTTG |
| heIF3-F | CGATTCGGTGATTGTAGTGG |
| heIF3-R | TTGTAGCCGTCGGCGTTC |
| IGF2BP2-F | CACTTCTCAGGCCAGACAGA |
| IGF2BP2-R | TGCTTCAGAAGTCCCCTCTG |
| hGAPDH-F | GAAGGTGAAGGTCGGAGTC |
| hGAPDH-R | GAAGATGGTGATGGGATTTC |
| **Primers for m6A-IP-qRT-PCR** |  |
| EEF1A2-peak-F | GGCAAGGAGAAGACCCACAT |
| EEF1A2-peak-R | CGCCTCCTTCTCGAACTTCT |
| IGF2BP2-peak-F | CTTTTCAGCAAGGCCTCTGG |
| IGF2BP2-peak-R | TACTAGTGAGCTTCCCGTGG |
| NUDCD1-peak-F | CCTGGAAAGCGATGGAGGT |
| NUDCD1-peak-R | TCTCGAAGTTTTACCTCTGCC |
| CRTC3-peak-F | GGAAAGCTCACCTCCACTG |
| CRTC3-peak-R | GGACGGAGATCAGTGGTTCT |
| DDX23-peak-F | ATTGCTCCCCAGACTGAACA |
| DDX23-peak-R | GTCTCCTGGGGCATCTAGG |
| UPF2-peak-F | GGGTCACATAATGCCAGCTG |
| UPF2-peak-R | CTCTCCTTGCTGCTCACTGT |
| PTPN21-peak-F | TCGTCCAACCCTAGCATCAC |
| PTPN21-peak-R | ATAGTCTGGGGTGGGGCG |
| ZNF703-peak-F | TGCGGAATCCACACACTTTG |
| ZNF703-peak-R | GGGTGGAGGAAGAGCTGTAG |
| hFOXO3-peak-F | CACCTTTGTGAATCCCACG |
| hFOXO3-peak-R | TCTGAATGAGGGTGGGTGG |
| hFOXO3-non-peak-F | CGTGCCTTGTCGAATTCTGT |
| hFOXO3-non-peak-R | AGGGTTTGCATAGACTGGCT |
| **Primers for plasmid construction** |  |
| FOXO3-CEK-F | GAGAGACTCGAGAGGATCACTGAGGAAGGGGAAGTGG |
| FOXO3-CEK-R | GAGAGAGCGGCCGCGGCAATGAGTGGAGAGCTGAGCTG |
| **Primers for fusion PCR** |  |
| FOXO3-mut-F | GCTCTCAGCAAGTGGATAGTGATACCGTTTAC |
| FOXO3-mut-R | GTAAACGGTATCACTATCCACTTGCTGAGAGC |
