# Supplementary material for: Altered m6A modification is involved in up‐regulated expression of FOXO3 in luteinized granulosa cells of non‐obese polycystic ovary syndrome patients
Source: J Cell Mol Med. 2020 Sep 1;24(20):11874–82. doi: 10.1111/jcmm.15807 (PMC7578862; doi:10.1111/jcmm.15807)
Supplement: Supplementary file 6 — Table S2 [file JCMM-24-11874-s006.docx]

**Supplemental Table S2.** **Sequences of the siRNA.**

| **Names of the siRNA** | **Sequence (5’→3’)** |
| --- | --- |
| YTHDF2-siRNA-1 | CCUACCAGAUGCAAUGUUUTT  AAACAUUGCAUCUGGUAGGTT |
| YTHDF2-siRNA-2 | CCAGCUUUCAGUCCAGCAATT  UUGCUGGACUGAAAGCUGGTT |
| METTL3-siRNA-1 | GCACUUGGAUCUACGGAAUTT  AUUCCGUAGAUCCAAGUGCTT |
| METTL3-siRNA-2 | GCAGAACAGGACUCGACUATT |
|  | UAGUCGAGUCCUGUUCUGCTT |
| METTL14-siRNA-1 | GGAUGAAGGAGAGACAGAUTT  AUCUGUCUCUCCUUCAUCCTT |
| METTL14-siRNA-2 | GCAGCACCUCGAUCAUUUATT  UAAAUGAUCGAGGUGCUGCTT |
| FTO-siRNA-1 | GGGUGUGAUAAGUGUGGAGTT  CUCCACACUUAUCACACCCTT |
| FTO-siRNA-2 | CCAGGUUGAUAAGGCACAATT  UUGUGCCUUAUCAACCUGGTT |
| ALKBH5-siRNA-1 | GCUAUGCUUCAGAUCGCCUTT  AGGCGAUCUGAAGCAUAGCTT |
| ALKBH5-siRNA-2 | GCUUCAGCUCUGAGAACUATT |
|  | UAGUUCUCAGAGCUGAAGCTT |
| Negative control | UUCUCCGAACGUGUCACGUTT  ACGUGACACGUUCGGAGAATT |
