# Supplementary material for: Altered m6A modification is involved in up‐regulated expression of FOXO3 in luteinized granulosa cells of non‐obese polycystic ovary syndrome patients
Source: J Cell Mol Med. 2020 Sep 1;24(20):11874–82. doi: 10.1111/jcmm.15807 (PMC7578862; doi:10.1111/jcmm.15807)
Supplement: Supplementary file 7 — Table S3 [file JCMM-24-11874-s007.docx]

**Supplemental Table S3. Clinical parameters in normovulatory women and PCOS patients.**

| **Variable** | **Controls** | **PCOS patients** |
| --- | --- | --- |
| **N** | 43 | 36 |
| **Age(yr)** | 28.00 ± 3.39 | 29.78 ± 2.91 |
| **BMI (kg/m^2^)** | 22.25 ± 3.31 | 24.61 ± 2.53 |
| **AMH（ng/ml）** | 3.89 ± 3.57 | 10.65 ± 4.31^a^ |
| **AFC** | 17.33 ± 3.67 | 34.00 ± 3.04 ^a^ |
| **LH (IU/L)** | 4.83 ± 1.32 | 5.28 ± 1.96 |
| **FSH (IU/L)** | 7.52 ± 1.75 | 6.67 ± 1.65 |
| **T (nmol/L)** | 1.34 ± 0.58 | 2.63 ± 0.49^a^ |
| **E2 (pmol/L)** | 164.92 ± 75.03 | 172.44 ± 58.26 |
| **PRL (mIU/L)** | 271.47 ± 57.18 | 347.00 ± 87.84 |

BMI, Body mass index. AFC, Antral follicle count. PRL, Prolactin. ^a^*P*<0.05 vs the control.
